# Multi-Temporal Prediction of High-Catch Fishing Grounds for Chub Mackerel (*Scomber japonicus*) Based on Deep Forest and SHapley Additive exPlanations (SHAP) of Environmental Contributions

**DOI:** 10.3390/biology15131031

**Published:** 2026-06-28

**Authors:** Leilei Zhang, Wei Fan, Fenghua Tang, Shenglong Yang, Yongchuang Shi, Shengmao Zhang

**Affiliations:** 1 East China Sea Fisheries Research Institute, Chinese Academy of Fishery Sciences, Shanghai 200090, China; m240752013@st.shou.edu.cn (L.Z.);; 2 College of Information and Technology, Shanghai Ocean University, Shanghai 200090, China; 3 Key Laboratory of Fisheries Remote Sensing, Ministry of Agriculture and Rural Affairs, Shanghai 200090, China

**Keywords:** Chub Mackerel, high-catch fishing grounds, Deep Forest, multi-temporal-scale fusion features, SHAP, nested spatial cross-validation, marine environmental factors, Northwest Pacific Ocean

## Abstract

Chub Mackerel (*Scomber japonicus*) is an important pelagic fishery resource in the Northwest Pacific, and its fishing-ground distribution is strongly influenced by dynamic marine environmental conditions. This study aimed to evaluate how environmental information at different temporal scales affects the prediction of high-catch fishing grounds and to identify environmental-variable contributions. Fishery logbook data from Chinese light purse seine vessels during 2014–2022 were combined with marine environmental variables to construct four feature sets: instantaneous features (E1), multi-temporal-scale fusion features (E2), short-term features with 7-day rolling means (E3), and long-term features with 30-day rolling means (E4). Deep Forest, random forest, XGBoost, LightGBM, and CatBoost were evaluated using nested spatial group cross-validation, and SHapley Additive exPlanations (SHAP) was applied to interpret model predictions. The results showed that, after historical environmental information was added, AUC values increased for most models, and the multi-temporal-scale fusion features performed better in metrics related to high-catch sample identification; therefore, the hypothesis proposed in this study was supported in the overall trend. Model comparisons showed that Deep Forest performed relatively stably under E2, E3, and E4, whereas RF performed relatively well under E1. Short-term environmental features helped improve overall fishing-ground discrimination, whereas multi-temporal-scale fusion was more favorable for identifying high-catch samples. Time-lag correlation and SHAP analyses indicated that short-term environmental changes, longer-term background conditions, and seasonal signals jointly provided information for model prediction. This study may provide a reference for real-time fishing-ground prediction and fishery management.

## Simple Summary

Chub Mackerel (*Scomber japonicus*) is an important fishery resource in the Northwest Pacific Ocean, but its distribution changes greatly with seasons and ocean conditions. For fishers and managers, predicting high-catch fishing grounds can help improve fishing efficiency and support more sustainable use of the resource. This study examined whether ocean information at different temporal scales can provide useful information for identifying high-catch fishing grounds. We considered instantaneous environmental conditions, short-term environmental changes, long-term environmental background, and the fusion of short-term and long-term information. The results showed that historical environmental information can partially compensate for the limitations of instantaneous environmental variables. Seven-day rolling environmental features helped improve overall model discrimination, whereas the integration of multi-scale environmental information was more favorable for identifying high-catch samples. SHAP and lag analyses indicated that model prediction jointly used environmental variables such as Chl-a, SST, SSS, DO, and MLD, together with their rolling statistics. These findings indicate that the prediction of high-catch fishing grounds for Chub Mackerel does not primarily depend on ocean conditions at a single time point, but is jointly associated with multi-temporal information, including instantaneous environmental conditions, short-term fluctuations, long-term background conditions, and seasonal signals. This study may support the development of more reliable fishing-ground prediction tools and decision-making in fishery production and resource management.

## 1. Introduction

As a typical pelagic migratory economic fish species, Chub Mackerel is widely distributed in the Northwest Pacific Ocean [1]. This species exhibits strong schooling behavior and seasonal migration [2]. Its spatial distribution is closely related to oceanographic conditions. As an important commercial fishery resource, Chub Mackerel plays a key role in regional fishery economies and provides stable production for coastal fisheries.

After Chinese light purse seine fisheries entered this area in 2014, the catch of Chub Mackerel showed clear interannual fluctuations. From 2014 to 2018, annual production remained at a relatively high level of approximately 110,000–130,000 tons, followed by a staged decline after 2019. In recent years, production has gradually recovered and stabilized at around 100,000 tons [3]. With increasing exploitation intensity and growing resource-management needs, the North Pacific Fisheries Commission (NPFC) has developed conservation and management measures for Chub Mackerel, requiring existing fishing members to avoid expanding their fleets and to implement monthly catch reporting [4]. In the context of large-scale fishery development, marked resource fluctuations, and strengthened regional management, systematically predicting high-catch fishing grounds and evaluating the contribution of environmental information at different temporal scales to prediction results are important for fishing operations, resource conservation, management decision-making, and sustainable use.

The distribution of Chub Mackerel fishing grounds is characterized by marked spatiotemporal dynamics and is closely associated with multiple marine environmental conditions. Fishing-ground prediction should not rely only on instantaneous environmental conditions but should also consider the predictive information that may be provided by physical environmental variability, seasonal background, and fish behavioral changes at different temporal scales. Therefore, evaluating the contribution of environmental information to fishing-ground prediction from a multi-temporal-scale perspective is important for improving prediction accuracy, fishing efficiency, and sustainable fishery-resource use.

In recent years, machine-learning methods have been widely used in fishing-ground prediction because of their strong nonlinear fitting ability and potential to improve predictive performance. However, existing studies still need improvement in feature construction, model interpretation, and model evaluation. Regarding feature construction, most previous studies have relied mainly on environmental variables at a single temporal scale, with limited systematic comparisons of environmental information across different time windows. Fishery-resource fluctuations are affected not only by instantaneous environmental conditions but also by seasonal, interannual, and longer-term oceanographic processes [5]. Primary production and its transfer through the food chain may also lead to delayed responses of fish populations to environmental change [6]. Therefore, modeling based only on a single temporal scale may fail to fully use multi-temporal information in the marine environment and its potential lagged associations.

For Chub Mackerel in the Northwest Pacific, previous studies have explored potential habitat prediction and fishing-ground forecasting. For example, Xue et al. [7] used a maximum entropy (MaxEnt) model with monthly environmental data to predict potential habitat distribution, and Han et al. [8] developed a central fishing-ground prediction model based on marine environmental variables and deep learning. These studies provide important references for understanding the relationship between Chub Mackerel distribution and the marine environment. However, they were generally based on predefined temporal scales or synchronous environmental features, and the comparison of multi-temporal environmental information and the characterization of lag effects remain limited. Zhou et al. [9] pointed out that fishery models are highly sensitive to spatial and temporal scale selection, and traditional studies often reflect resource–environment relationships under specific scale settings. Therefore, incorporating multi-temporal-scale environmental features may help to more comprehensively assess the association between environmental features and fish distribution predictions.

In terms of model interpretation, although machine-learning methods have achieved high predictive accuracy in fishing-ground prediction, their black-box nature remains an important limitation for practical application. In recent years, SHAP has been introduced into environmental and ecological modeling to improve interpretability [10]. SHAP has been widely applied in climate-change modeling [11] and fishery-resource prediction [12]. Lu et al. [13] combined SHAP with multiple machine-learning algorithms to evaluate the contributions of environmental variables to classification results. In studies of tuna and squid, SHAP has been used to identify the contributions of SST, DO, and SSS to fishing-ground or spatial-distribution prediction, thereby helping interpret the associations between environmental variables and model prediction results [14,15]. These studies show that SHAP can enhance model interpretability and provide detailed information on variable importance and sensitivity.

In model evaluation, many studies still use random splitting of training and test sets without fully considering spatial autocorrelation, which may lead to overestimated model performance [16,17]. By contrast, spatial-block cross-validation can more effectively evaluate model generalization to unknown areas and improve the robustness and credibility of performance estimates [18].

Although previous studies have examined the relationship between Chub Mackerel fishing-ground distribution and the marine environment from different perspectives, most existing studies have used environmental variables at a single temporal scale, and the role of environmental information at different temporal scales in high-catch fishing-ground prediction remains insufficiently evaluated. Therefore, the core research question addressed in this study is: how does environmental information at different temporal scales affect the prediction performance for high-catch fishing grounds of Chub Mackerel? Based on this research question, we propose the following hypothesis: compared with using only instantaneous environmental variables, adding historical environmental information can improve the prediction ability for high-catch fishing grounds of Chub Mackerel (defined based on catch).

Therefore, the main objective of this study is to evaluate how environmental information at different temporal scales affects the prediction of high-catch fishing grounds for Chub Mackerel (defined based on catch). Around this objective, this study constructed a multi-temporal environmental feature system including instantaneous (E1), short-term (E3), long-term (E4), and multi-scale fusion (E2) feature sets. E1 consists of daily environmental variables and monthly cyclic terms to describe current environmental conditions and instantaneous responses. E3 adds a 7-day rolling mean to E1 to capture weekly environmental fluctuations and potential lag effects. E4 adds a 30-day rolling mean to E1 to characterize the monthly environmental background and long-term cumulative effects. E2 integrates both 7-day and 30-day rolling means to examine the synergistic representation ability of multi-temporal environmental information. Subsequently, Deep Forest was compared with random forest (RF), eXtreme Gradient Boosting (XGBoost), Light Gradient Boosting Machine (LightGBM), and CatBoost. To ensure rigorous and reliable evaluation, a spatially grouped nested cross-validation framework was used for model training and hyperparameter optimization, and SHAP was used to interpret the contributions of environmental variables to model prediction. The main results indicate that historical environmental information can complement instantaneous environmental variables and help improve model prediction performance; 7-day rolling features help improve overall discrimination, whereas multi-temporal-scale fusion is more favorable for identifying high-catch samples. SHAP and lag analyses further indicate that model prediction jointly uses multi-temporal environmental information, including Chl-a, SST, SSS, DO, MLD, and their rolling statistics. This study may provide a reference for near-real-time fishing-ground prediction and fishery management.

## 2. Materials and Methods

### 2.1. Study Area and Data Sources

**Figure 1:**
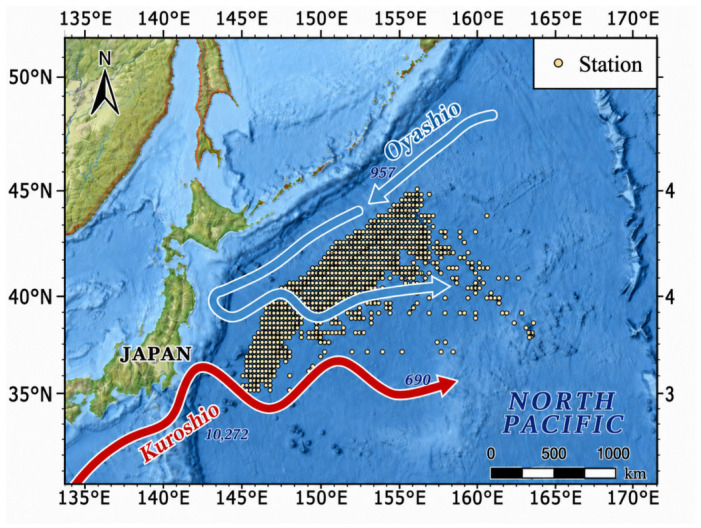
Study area, fishing-sample distribution, and major ocean-current background in the Northwest Pacific.

The fishery data used in this study were derived from catch logbooks of Chinese light purse seine vessels targeting Chub Mackerel from April to December during 2014–2022. The study area is located approximately between 145° E–163° E and 35° N–45° N in the high seas of the Northwest Pacific, near the Kuroshio Extension and the Oyashio-influenced region (Figure 1). The dataset contains 70,147 fishing records. Each record includes catch, operation time, longitude, latitude, and matched environmental variables. The temporal resolution was daily, and environmental variables were matched to fishing records on a 0.5° × 0.5° spatial grid.

**Figure 2:**
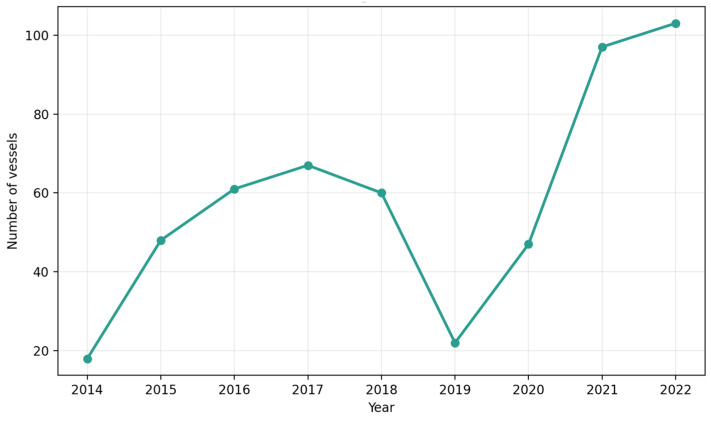
Changes in the number of Chinese light purse seine vessels during 2014–2022.

Figure 2 shows changes in the number of Chinese light purse seine vessels from 2014 to 2022. The number of vessels increased rapidly from 2014 to 2017, declined markedly during 2018–2019, and then increased again after 2020, reflecting staged fluctuations in fishing intensity during the study period.

**Figure 3:**
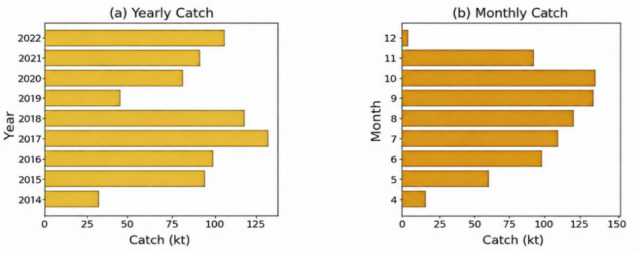
Annual total catch (**a**) and monthly catch (**b**) of Chub Mackerel from 2014 to 2022.

Figure 3 shows the annual total catch and accumulated monthly catch during 2014–2022. The annual catch varied among years, with higher catches during 2017–2018 and relatively low levels in 2014 and 2019. Monthly catches were mainly concentrated from June to November, with higher catches in September and October. This temporal pattern provides a basis for introducing monthly cyclic variables and multi-temporal features.

Marine environmental data were obtained from the Copernicus Marine Service (https://resources.marine.copernicus.eu/products (accessed on 1 March 2026)) and matched to the temporal and spatial resolution of the fishery logbooks. The environmental variables included sea surface temperature (SST), sea surface salinity (SSS), chlorophyll-a concentration (Chl-a), sea level anomaly (SLA), current velocity (CV), dissolved oxygen (DO), and mixed layer depth (MLD). CV was calculated from the zonal geostrophic velocity (UGOS) and meridional geostrophic velocity (VGOS) as

(1) $$\begin{array}{c} CV=\sqrt{{UGOS}^{2}+{VGOS}^{2}} \end{array}$$

UGOS and VGOS represent zonal and meridional flow components, respectively. Their vector synthesis provides a simplified measure of current intensity and is suitable for large-scale ocean-circulation and fishing-ground distribution modeling. This treatment simplifies the model structure while retaining an interpretable representation of flow strength.

A binary classification framework was adopted. The median daily catch was used as the threshold: areas with catches equal to or above the median were defined as high-catch fishing grounds, whereas the remaining records were defined as low-catch fishing grounds. The dataset contained 35,075 high-catch samples and 35,072 low-catch samples, indicating a nearly balanced class distribution. Accordingly, model performance was evaluated using AUC, F1-score, precision, recall, and PR-AUC.

### 2.2. Construction of Multi-Temporal Features

To characterize the predictive role of environmental information at different temporal scales, four feature sets were constructed using the same preprocessing, spatial matching, and labeling framework (Table 1). Previous studies have shown that species distribution and fishing-ground prediction are temporally scale-dependent, and environmental variables at different temporal scales may provide different predictive information [19]. In addition, fishing-ground prediction studies have compared environmental windows from daily to monthly scales [20]. Therefore, this study selected 7-day and 30-day rolling means as predefined operational time windows to represent weekly short-term environmental changes and monthly environmental background conditions, respectively, rather than variable-specific optimal windows determined by subsequent lag-correlation analysis. E1, the instantaneous feature set, includes daily environmental variables and monthly cyclic terms (month_sin and month_cos) to describe current oceanographic conditions and seasonal signals. E3 adds the 7-day rolling means of environmental variables to E1 to represent weekly fluctuations and potential lag effects. E4 adds the 30-day rolling means to E1 to describe monthly trends and long-term cumulative effects. E2 includes both 7-day and 30-day rolling means to evaluate the synergistic representation of short- and long-term environmental information.

Rolling statistics were calculated using a past-only window. Only historical observations before time t were used; the current day and future information were excluded to avoid information leakage and maintain temporal causality. The 7-day rolling mean was calculated from t − 7 to t − 1, and the 30-day rolling mean was calculated from t − 30 to t − 1. Rolling features were computed prior to cross-validation splitting, based solely on the gridded environmental time series at each 0.5° × 0.5° cell, without incorporating any catch labels or test-set information. The resulting rolling features were then matched to the fishery logbook records by date and grid cell, after which k-means spatial grouping and nested spatial GroupKFold were applied. This procedure ensures that no label leakage or model-selection leakage occurs across cross-validation folds. Subsequent experiments changed only the feature-input structure while keeping the model configuration and validation protocol consistent, thereby ensuring interpretability and fairness in comparisons among feature groups.

This study employs sine and cosine transformations to map months onto a unit circle, where m denotes the month, ranging from 1 to 12. Equations (2) and (3) generate $month_{sin}$ and ${month}_{cos}$ respectively, mapping months onto the unit circle to preserve cyclic adjacency and jointly represent intra-annual seasonal phase information [21]:

(2) $${month}_{\sin}=\sin\left(\frac{2\pi m}{12}\right)$$

(3) $${month}_{\cos}=\cos\left(\frac{2\pi m}{12}\right)$$

This encoding preserves the circular topology of monthly seasonality, ensuring that adjacent months remain close in continuous space and avoiding artificial linear distances introduced by integer month coding. The orthogonal sine and cosine components jointly represent seasonal phase information and are suitable for describing seasonal changes in marine environments.

#### Time-Lag Analysis

Considering that marine ecological and physical processes usually involve certain delayed effects, the influence of different environmental factors on fishing-ground formation may not occur synchronously. To further examine whether potential lagged associations exist between environmental variables and the distribution of high-catch fishing grounds defined based on catch, this study conducted a post hoc time-lag diagnostic analysis after the predefined multi-temporal feature sets had been constructed. It should be noted that this analysis was not used as the basis for feature construction, but served as an exploratory diagnostic method to help interpret the possible temporal association characteristics of different environmental variables.

For each environmental variable, a time-lag window of 0–30 days was set. For any given lag time l, the environmental variable at time t−l was paired with the target variable at time t, and their correlation was calculated to characterize the delayed response relationship between environmental changes and fishing-ground occurrence. The correlation was measured using Spearman’s rank correlation coefficient [22], calculated as follows:

(4) $$\begin{array}{c} r_{s}\left(l\right)={corr}_{s}\left(X_{t-l},Y_{t}\right) \end{array}$$

where $X_{t-l}$ represents the environmental variable lagged by l days, $Y_{t}$ represents the target variable at time t, and $r_{s}(l)$ denotes the correlation coefficient corresponding to the given lag time. Spearman’s rank correlation coefficient was used in the time-lag diagnostic analysis to explore potential lagged monotonic associations between environmental variables and high-catch labels defined based on catch. Compared with Pearson’s correlation, Spearman’s correlation is a rank-based non-parametric method with fewer assumptions regarding variable distribution and linearity, making it suitable for the exploratory diagnostic analysis in this study. This analysis was not used for parametric hypothesis testing or causal inference, but only to assist in identifying variable-specific temporal association characteristics.

### 2.3. Research Workflow

**Figure 4:**
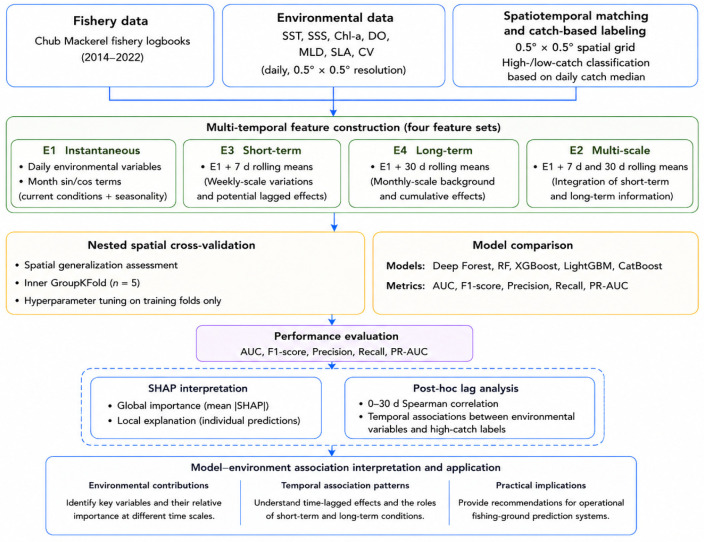
Overall research workflow.

The overall workflow is shown in Figure 4. In this figure, arrows indicate the sequential workflow of the study, and the dashed box denotes the interpretability and post-hoc lag analyses conducted after model performance evaluation. Fishery logbook records and marine environmental data from 2014 to 2022 were used as the data basis, and the median daily catch was used to divide samples into binary high-catch and low-catch labels. Four environmental feature sets were then constructed based on predefined time windows to represent instantaneous, short-term, long-term, and multi-temporal environmental information. Deep Forest, RF, XGBoost, LightGBM, and CatBoost were trained, optimized, and evaluated under a spatially grouped nested cross-validation framework. Performance was assessed using AUC, F1-score, Precision, Recall, and PR-AUC. Finally, SHAP was used to analyze the contributions of environmental variables at global and local levels, and 0–30-day lag-correlation analysis was combined to help interpret temporal association characteristics between environmental variables and high-catch prediction.

### 2.4. Deep Forest and Other Machine-Learning Models

Deep Forest, also known as gcForest, is a hierarchical ensemble-learning model proposed by Zhou and Feng [23]. It learns new feature representations layer by layer through a cascade of forests, thereby providing hierarchical representation learning similar to deep neural networks. Unlike a traditional single-layer random forest, Deep Forest stacks multiple forests in a cascade structure and progressively enhances feature representation across layers, which helps model complex feature relationships and potential interactions.

The core idea of Deep Forest is a cascade forest architecture. Each layer consists of multiple random forests or completely random forests. The input features are first passed to the first-layer forest models, which output class-probability vectors. These probability vectors are concatenated with the original features and used as the input to the next layer. Through this layer-wise cascade, the model continuously generates higher-level feature representations. Training proceeds layer by layer, and the model stops growing when adding a new layer does not improve validation performance. This adaptive-depth mechanism allows model complexity to be adjusted according to data complexity and helps reduce overfitting risk.

Because Deep Forest adopts a hierarchical feature-enhancement structure, it is theoretically suitable for multi-scale or multi-source features. In the cascade structure, features from different scales can be combined and reconstructed layer by layer, which may help capture nonlinear interactions. Previous studies have shown that Deep Forest can continuously generate new feature representations in complex feature spaces and improve the representation ability and prediction stability of multidimensional data [24].

RF, XGBoost, LightGBM, and CatBoost were selected as comparison models. RF, proposed by Breiman in 2001, is based on bagging and random feature selection and is known for stability and ease of use [25]. XGBoost, released in 2016, is widely used because of its ability to handle sparse data, regularization, and parallel computation [26]. LightGBM is a gradient boosting decision tree framework that uses leaf-wise growth, gradient-based one-sided sampling, and exclusive feature bundling, making it suitable for large-scale modeling. CatBoost, developed by Yandex, provides efficient categorical-feature handling and ordered boosting to reduce information leakage [27].

Deep Forest is also based on tree ensembles but builds a multilayer learning framework through cascaded forests. Compared with single-layer ensemble models such as RF, Deep Forest can continuously enhance feature representation through multiple layers and capture complex nonlinear relationships. Compared with gradient boosting models such as XGBoost, LightGBM, and CatBoost, which rely on iterative residual fitting, Deep Forest performs feature enhancement and ensemble learning through a cascade structure. Systematic comparison of Deep Forest with these mainstream ensemble models helps evaluate its performance and applicability for multi-temporal environmental feature modeling.

### 2.5. Experimental Design

#### 2.5.1. Variable Selection and Collinearity Testing

**Figure 5:**
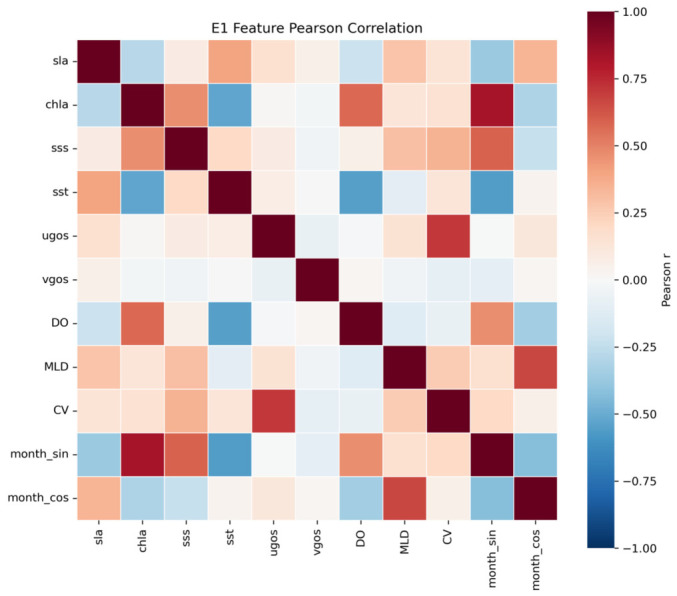
Pearson correlation matrix among variables.

**Figure 6:**
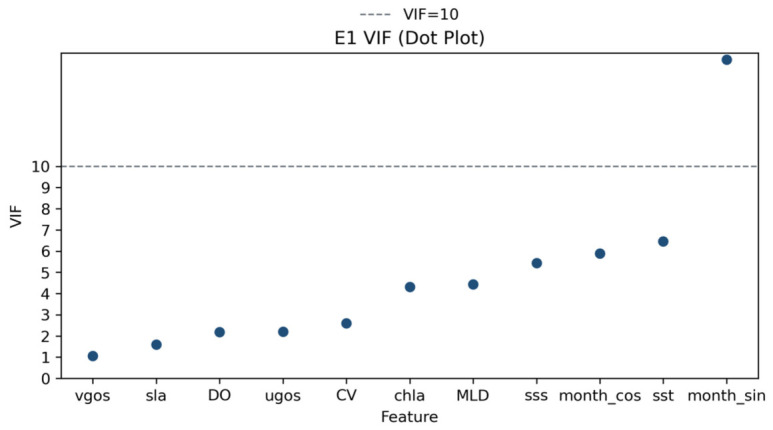
VIF results for features in E1.

Pearson correlation coefficients were calculated for all variables, and a correlation matrix was plotted to evaluate pairwise relationships and potential collinearity (Figure 5). Most variables showed weak correlations, and the absolute correlation coefficients of all variable pairs were below 0.9, a commonly used threshold for collinearity screening. Variance inflation factor (VIF) analysis was further conducted for the base feature set E1 (Figure 6). In Figure 6, the dashed line indicates the VIF threshold of 10, above which variables are generally considered to show potential multicollinearity. Most variables had VIF values below 10. Only the seasonal cyclic variables month_sin and month_cos were slightly higher because of their mathematical relationship as sine–cosine encodings. Because these two variables jointly represent the annual seasonal phase, both were retained for subsequent modeling. No severe multicollinearity was detected among environmental variables.

#### 2.5.2. Cross-Validation Protocol

A nested group cross-validation framework was used for model training and evaluation. The outer validation stage was used to estimate model generalization performance. GroupKFold with six splits was applied using spatial blocks as group labels, so that samples within the same spatial group did not appear simultaneously in the training and test sets, thereby reducing the risk of performance overestimation caused by spatial autocorrelation.

This design reduces the risk of overestimating performance due to spatial autocorrelation. The inner validation stage was used for hyperparameter selection and model selection. A five-fold GroupKFold was conducted only within the outer training data and did not involve the outer test data [28]. This nested structure prevents information leakage from hyperparameter tuning and improves the reliability of performance estimates. Spatial groups were generated using k-means clustering based on the longitude and latitude coordinates of fishing records and were used as group labels in GroupKFold, ensuring that samples from the same spatial group did not appear simultaneously in the training and test sets. It should be noted that no explicit buffer was set between training and test spatial groups in this study. Therefore, this spatial grouping strategy can reduce the risk of spatial leakage caused by random splitting, but it cannot completely eliminate fine-scale spatial autocorrelation near group boundaries.

#### 2.5.3. Hyperparameter Strategy

To ensure fair comparisons among different feature sets and models, hyperparameter optimization was performed separately for each model and each temporal-scale feature set (E1, E2, E3, and E4) under the nested spatial group cross-validation framework. Hyperparameter search was conducted only within the corresponding outer training set using five-fold GroupKFold, and the outer test set was used only for final performance evaluation and was not involved in any hyperparameter selection. Candidate configurations were randomly sampled from predefined parameter grids, and the hyperparameter search space for each model is summarized in Table 2. The mean inner-validation AUC was used as the main selection criterion. For each model-feature-set combination, the configuration with the highest mean AUC was selected as the optimal parameter configuration; the model was then retrained on the complete outer training set and evaluated on the corresponding outer test set. This strategy avoids potential bias caused by using a single feature set for unified tuning and allows each feature set to be independently optimized within the same validation framework, thereby improving the fairness of model and feature-set comparisons.

### 2.6. SHAP Interpretation Method

Since its proposal in 2017, SHAP has become an important method for interpreting machine-learning models and has been widely used for tree-based models such as XGBoost, LightGBM, RF, and CatBoost [29]. Because Deep Forest is also composed of multilayer decision-tree ensembles, its predictive process can be interpreted using SHAP to quantify the contribution of each environmental variable to model output and to analyze potential interactions among features. In this study, SHAP values were calculated to quantify the contributions of environmental variables to model prediction at different temporal scales and to analyze the associations between these variables and prediction results. The SHAP value is based on the Shapley value from cooperative game theory and represents the average marginal contribution of a feature to the model output. For model f, the SHAP values of the features are defined as follows:

(5) $$\phi_{i}\left(f,x\right)=\sum_{S\subseteq N\setminus \left\{i\right\}}\frac{\left|S\right|!\left(\left|N\right|-\left|S\right|-1\right)!}{\left|N\right|!}\left[f_{S\cup \left\{i\right\}}\left(x_{S\cup \left\{i\right\}}\right)-f_{S}\left(x_{S}\right)\right]$$

where N denotes the set of all features, S represents any subset of features that does not include feature i, and |S| denotes the number of features in subset S. $f_{S}(x_{S})$ represents the model prediction obtained using only the feature subset S. $\phi_{i}$ denotes the SHAP value of feature i, that is, the average marginal contribution of this feature to the model output.

## 3. Results

### 3.1. Multi-Model Comparison Across Temporal Scales

**Figure 7:**
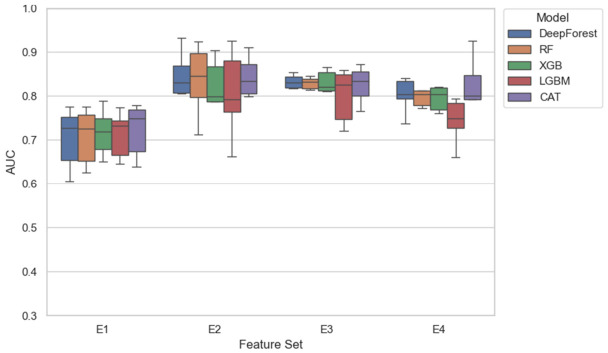
Outer-fold AUC distributions by feature set and model.

**Figure 8:**
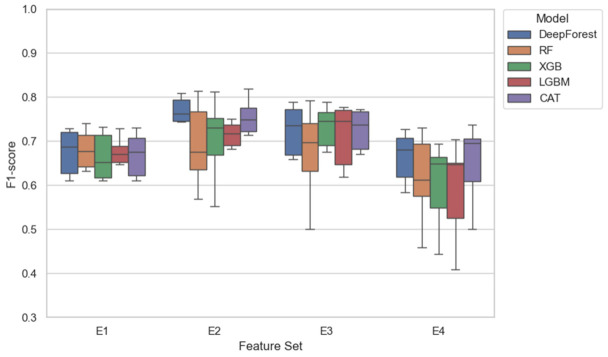
Outer-fold F1-score distributions by feature set and model.

Different temporal-scale feature configurations had certain effects on model performance (Table 3). Overall, the AUC and F1-score values of models under the E1 feature set were relatively low, indicating that using only instantaneous environmental variables may be insufficient to fully represent high-catch fishing-ground information defined based on catch. After rolling environmental statistics were introduced, the performance metrics of most models under E2, E3, and E4 increased numerically, indicating that historical environmental information may help improve fishing-ground prediction. For positive-class identification, the F1-score, Precision, and Recall of E2 were numerically higher, and Deep Forest in particular achieved relatively good overall numerical results under E2, suggesting that integrating 7-day and 30-day environmental statistics may help improve high-catch sample identification based on catch. The PR-AUC values of most models under E2 were also relatively high, further indicating that multi-temporal-scale features have certain advantages in positive-sample identification and ranking ability. The AUC values of E3 were also relatively high, suggesting that weekly environmental dynamics may help distinguish high-catch and low-catch states. By contrast, when E4 was used alone, metrics related to positive-class identification were relatively low, indicating that 30-day environmental background information alone contributed less. From the model perspective, Deep Forest achieved relatively high numerical performance under E2, E3, and E4, whereas RF performed relatively well under E1 and achieved the highest AUC under E2, suggesting that different models differed in their ability to use temporal-scale features. As shown in Table 4, this study comprehensively considered AUC, F1-score, Precision, Recall, and PR-AUC to select models with balanced performance in discrimination ability, positive-class identification, and prediction stability. Figure 7 and Figure 8 show the distributions of AUC and F1-score across outer cross-validation folds for each model under different temporal-scale feature sets, and the results are generally consistent with Table 3 and Table 4. Overall, short-term environmental dynamics and multi-temporal environmental information may both contribute to model prediction, with E2 showing relatively good numerical performance in high-catch sample identification.

### 3.2. Effects of Temporal Scale on Deep Forest

**Figure 9:**
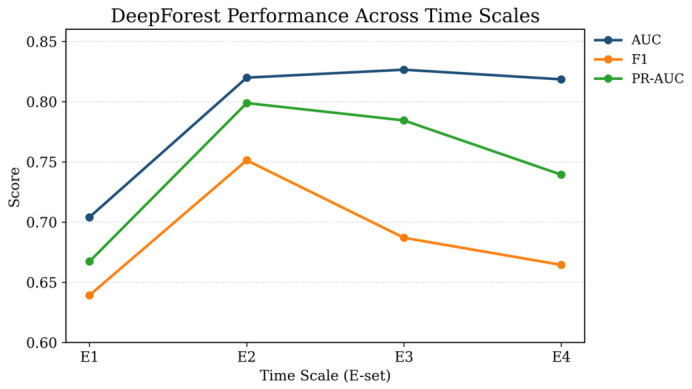
Performance comparison of Deep Forest under different feature sets.

Deep Forest showed certain numerical differences among different temporal-scale feature combinations (Figure 9). Compared with E1, which includes only instantaneous environmental variables, the introduction of temporal dynamic features generally increased the AUC, F1-score, and PR-AUC values under E2, E3, and E4, indicating that historical environmental information may complement instantaneous environmental variables and help improve Deep Forest-based fishing-ground prediction.

Deep Forest achieved a relatively high AUC value under E3, suggesting that 7-day rolling environmental features may help enhance the overall discrimination ability of the model for high-catch and low-catch states. E2 achieved relatively high values in F1-score and PR-AUC, indicating that integrating 7-day and 30-day environmental statistics may help improve high-catch sample identification and ranking ability based on catch. By contrast, the metrics of E4 were lower than those of E2 and E3 but still higher than those of E1, indicating that 30-day environmental background information has some predictive value, although its independent contribution to model improvement is relatively limited.

Ecologically, short-term environmental changes may be related to fish aggregation and foraging activity, and short-term features may provide more discriminative dynamic information [30]. Long-term environmental conditions reflect the background state of the marine ecosystem [31,32], change more slowly, and may have a weaker immediate influence on fishing-ground formation. When short- and long-term information are combined, variables at different temporal scales may complement each other and improve fishing-ground prediction. Deep Forest showed a certain response to short-term environmental dynamics and multi-temporal environmental information, among which E2 and E3 showed relatively good numerical performance.

### 3.3. Time-Lag Analysis Results

**Figure 10:**
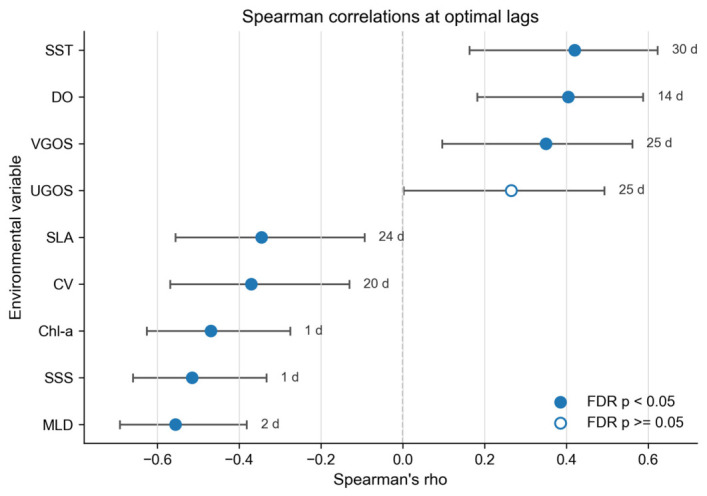
Spearman correlations between high-catch fishing grounds for Chub Mackerel and environmental variables at the optimal lag times.

Table 5 gives the optimal lag time, Spearman rank correlation coefficient, *p* value, FDR-corrected *p* value, and 95% confidence interval for each environmental variable across the candidate 0–30-day lag range. Figure 10 shows the Spearman correlation coefficient and its 95% confidence interval for each variable at the optimal lag time. This analysis is a post hoc diagnostic analysis and is used only to describe possible temporal association characteristics between environmental variables and high-catch fishing grounds defined based on catch; it is not used for feature construction or causal inference. The results showed differences among environmental variables in optimal lag time and correlation direction. Chl-a, SSS, and MLD had short optimal lags of 1 day, 1 day, and 2 days, respectively, and all showed negative correlations. DO and CV had optimal lags of 14 days and 20 days, respectively, showing moderate lagged associations. SLA, SST, UGOS, and VGOS had longer optimal lags of 24, 30, 25, and 25 days, respectively, suggesting that some ocean-dynamic and thermal environmental variables may be associated with high-catch fishing-ground distribution at longer temporal scales. Except for UGOS, all variables remained significant after FDR correction (*p* < 0.05). Overall, the lag analysis indicated variable-specific temporal association characteristics between environmental variables and high-catch fishing grounds defined based on catch.

### 3.4. Global SHAP Analysis of Cross-Scale Environmental-Variable Contributions

**Figure 11:**
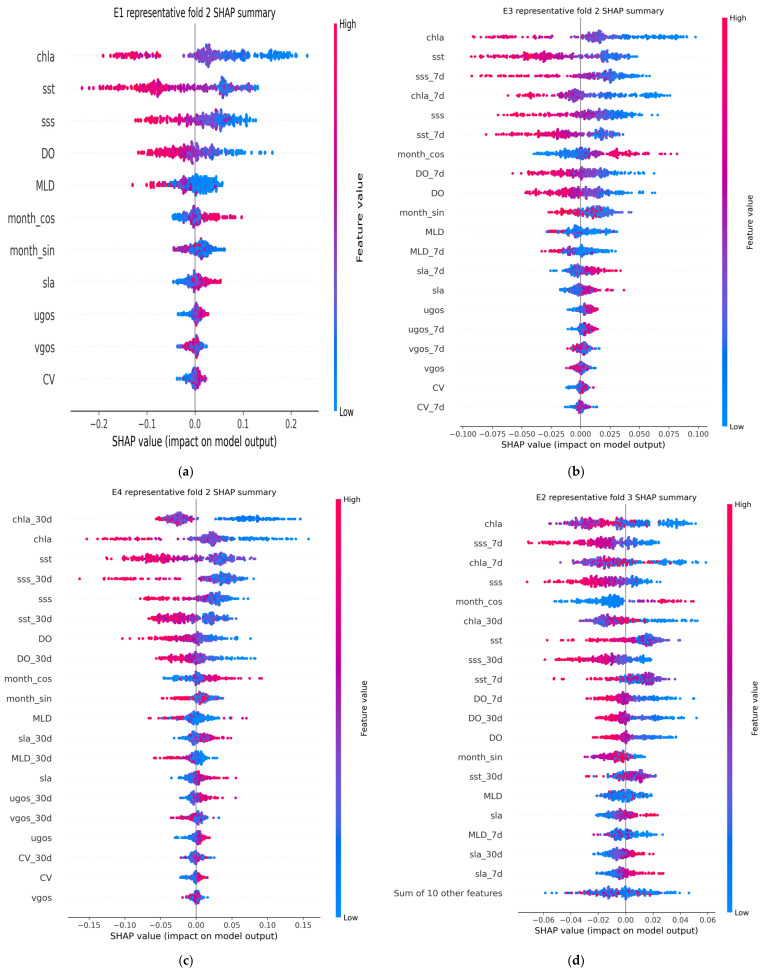
Global SHAP importance distributions of environmental variables under different temporal-scale feature sets: (**a**) E1, (**b**) E3, (**c**) E4, and (**d**) E2.

To interpret the contributions of environmental variables to model prediction at different temporal scales, this study conducted a global SHAP analysis. SHAP summary plots ranked variable importance in model prediction by mean absolute SHAP values and showed the contribution direction of variable values to model output (Figure 11). It should be noted that SHAP analysis mainly reflects the contributions of environmental variables during model prediction and can be used to understand the associations between variables and prediction results, but it does not directly represent causal relationships. In E1 (Figure 11a), Chl-a, SST, SSS, DO, and MLD ranked high, indicating that instantaneous environmental variables contributed substantially to model prediction. Among them, Chl-a and SST showed relatively wide SHAP distributions, suggesting that primary-production-related information and sea-surface temperature information were related to changes in model output. In E3 (Figure 11b), Chl-a, SST, SSS_7d, Chl-a_7d, and SSS ranked high, indicating that instantaneous variables and 7-day rolling variables jointly provided information for model prediction. The relatively high rankings of SSS_7d, Chl-a_7d, and SST_7d indicate that weekly environmental changes were associated with model prediction results. In E4 (Figure 11c), 30-day rolling variables contributed prominently. Chl-a_30d ranked highest, and SSS_30d, SST_30d, and DO_30d also entered relatively high positions, indicating that monthly environmental background information could be used by the model for prediction. These variables may provide information related to longer-term primary production, water-mass conditions, and thermal environmental background. In E2 (Figure 11d), instantaneous, 7-day, and 30-day rolling variables jointly contributed to model prediction. Chl-a, SSS_7d, Chl-a_7d, SSS, and month_cos ranked high, while Chl-a_30d, SSS_30d, SST_7d, DO_7d, and DO_30d also had certain contributions, indicating that the multi-temporal feature set could simultaneously provide current environmental states, short-term environmental changes, and longer-term environmental background information.

Overall, SHAP results showed that the environmental variables emphasized by the model differed among temporal scales. Instantaneous Chl-a, SST, and SSS had high contributions in multiple feature sets; 7-day rolling variables mainly provided short-term environmental information; and 30-day rolling variables provided longer-term environmental background. Seasonal variables, including month_cos and month_sin, also made certain contributions in some feature sets. Taken together, model predictions did not rely on a single environmental variable but integrated environmental information at different temporal scales.

### 3.5. Local SHAP Analysis of Environmental-Variable Contributions

For local interpretation, SHAP waterfall plots were used to visualize the prediction process for representative samples under different temporal feature configurations (E1–E4). The plots show the direction and magnitude of each feature contribution to the model output. Red indicates a positive contribution, blue indicates a negative contribution, and bar length represents the magnitude of the contribution.

The selected samples were correctly classified as high-catch and low-catch samples from the outer test set and were used to show local feature contributions under different prediction states. It should be noted that local SHAP results reflect only the model explanation process for representative samples and are not used to support general ecological causal inference.

**Figure 12:**
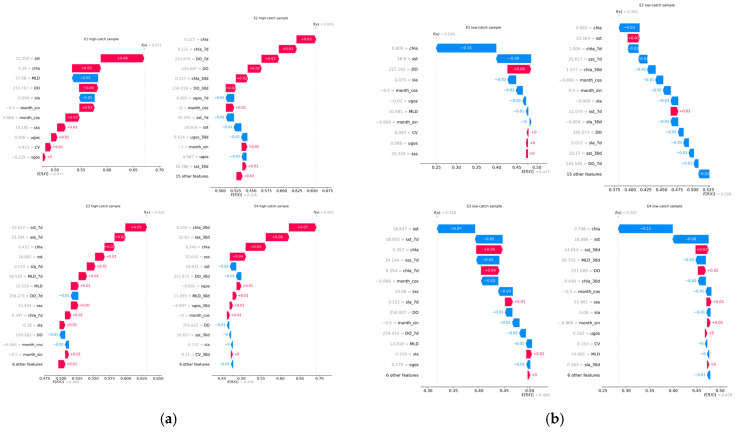
Local SHAP for representative high- and low-catch samples under different temporal-scale feature sets: (**a**) high-catch sample; (**b**) low-catch sample.

For the high-catch sample (Figure 12a), variables at different temporal scales all made local contributions to the model output. In E1, instantaneous SST, Chl-a, and DO made positive contributions to the high-catch prediction output, whereas MLD and SLA made negative contributions, indicating that the model mainly adjusted the prediction result according to the current environmental conditions of this sample. In E2, instantaneous Chl-a, Chl-a_7d, DO_7d, DO, Chl-a_30d, and DO_30d all made positive contributions, indicating that the model simultaneously used current, short-term, and longer-term environmental information in this sample. In E3, SST_7d, SSS_7d, Chl-a, SST, SLA_7d, and MLD_7d jointly contributed to the model output, indicating that 7-day rolling variables participated in the local prediction for this sample. In E4, Chl-a_30d, SSS_30d, Chl-a, and SSS made prominent contributions, indicating that both 30-day rolling variables and instantaneous variables were used by the model to predict this high-catch sample.

For the low-catch sample (Figure 12b), the main variables mostly contributed to reducing the high-catch prediction output. In E1, Chl-a and SST made large negative contributions to the model output, causing the predicted value of this sample to fall below the baseline output. In E2, Chl-a, Chl-a_7d, SSS_7d, Chl-a_30d, month_cos, and month_sin all made negative contributions, indicating that instantaneous, short-term, and longer-term variables all participated in the local identification of this low-catch sample. In E3, SST, SST_7d, SSS_7d, month_cos, and SSS reduced the high-catch prediction output. In E4, Chl-a, SST, Chl-a_30d, MLD_30d, month_cos, and SLA overall reduced the model output, indicating that instantaneous variables and 30-day rolling variables jointly participated in the prediction of this low-catch sample.

Local SHAP results were generally consistent with the global SHAP analysis. Chl-a, SST, SSS, and their rolling statistics showed certain local contributions in multiple representative samples, indicating that these variables not only ranked high in global importance but also participated in the model prediction process for individual samples. At the same time, 7-day and 30-day rolling variables appeared in the local explanations for E2, E3, and E4, further indicating that the model could use environmental information at different temporal scales. It should be noted that local SHAP plots only reflect the prediction process of selected samples and should not be directly generalized as general ecological mechanism explanations.

## 4. Discussion

### 4.1. Effects of Multi-Temporal Environmental Features on Model Performance

This study aimed to evaluate the effects of environmental information at different temporal scales on the prediction of high-catch fishing grounds for Chub Mackerel, defined based on catch. The main results showed that E3 had a relatively high AUC value, suggesting that 7-day rolling environmental features may help improve the overall discrimination ability of the model for high-catch and low-catch states. E2 performed relatively well in positive-class identification metrics, including F1-score, Precision, Recall, and PR-AUC, indicating that simultaneously integrating 7-day and 30-day environmental statistics may be more favorable for identifying high-catch samples. Lag analysis also showed that Chl-a, SSS, and MLD reached optimal lags within 1–2 days, indicating possible short-time-scale statistical associations between these variables and high-catch fishing grounds. By contrast, when E4 was used alone, F1-score and Recall values were relatively low, suggesting that 30-day environmental background information had limited supplementary value for positive-class identification and was more suitable as background information in multi-temporal-scale features.

These results indicate that the prediction of high-catch fishing grounds for Chub Mackerel is not related only to environmental conditions within a single time window, but is jointly associated with multi-temporal information such as short-term environmental fluctuations and long-term background conditions.

Elith and Leathwick [33] noted that species distribution prediction has strong temporal-scale dependence and that different ecological processes correspond to different predictable time windows. Existing Chub Mackerel studies also indicate clear spatiotemporal dynamics in fishing-ground distribution. Li et al. [34] showed that the effects of marine environmental factors on Chub Mackerel distribution have strong spatiotemporal heterogeneity. Zhao et al. [35] analyzed the spatiotemporal distribution, population dynamics, and resource status of Chub Mackerel in the high seas of the Northwest Pacific using commercial fishery data and emphasized the importance of understanding its spatiotemporal distribution for commercial fishing and fishery management. Compared with previous studies, this study further refined the analysis from the perspective of environmental-feature time windows and showed that different temporal windows contributed differently to high-catch fishing-ground prediction.

Similar temporal-scale sensitivity has been reported for other pelagic or oceanic migratory species. Xie et al. [36] showed in squid fishing-ground prediction that short-term environmental windows can capture rapidly changing processes such as fronts and eddies, and that appropriate short- to medium-term spatiotemporal scales can improve fishing-ground prediction. This is consistent with the relatively high AUC value of E3 in this study and suggests that short-term environmental changes often provide sensitive discriminative information for schooling and migratory pelagic species.

In addition to environmental time windows, seasonal-phase information may also contribute to improved model prediction performance. In this study, month_sin and month_cos were used to encode the month cyclically and represent the intra-annual seasonal phase. SHAP results showed that seasonal variables still made certain contributions in some feature sets, especially month_cos, which ranked relatively high in E2, indicating that seasonal-phase information may provide background information for multi-temporal prediction. Previous studies have shown that the distribution, spawning suitability, and resource changes in Chub Mackerel have clear seasonal characteristics. Wang et al. [37] developed a temperature suitability index for Chub Mackerel during the spawning season and found that climate change may alter spawning-environment suitability and further affect resource changes. Therefore, the contributions of month_sin and month_cos in the model may be related to seasonal migration, intra-annual fishing-ground changes, and the seasonal distribution of fishing operations, but this interpretation remains at the level of predictive association.

It should be noted that the contribution of seasonal variables does not mean that the model mainly relies on month information, nor does it imply that seasonal variables can replace specific environmental variables. Instead, seasonal variables mainly provide intra-annual temporal background, whereas environmental variables and their rolling statistics further characterize marine environmental information under specific temporal and spatial conditions. Combining lag analysis with SHAP results shows that short-term variables and their rolling statistics provided relatively sensitive information for model prediction, whereas long-term variables and seasonal variables provided more background information. Overall, the prediction of high-catch fishing grounds for Chub Mackerel was associated with both multi-temporal environmental information and seasonal-phase background, but it should not be directly interpreted as an ecological causal mechanism.

### 4.2. Analysis of Model-Performance Differences

This study compared Deep Forest, RF, XGBoost, LightGBM, and CatBoost under multi-temporal environmental features. In E1, which contained only instantaneous environmental variables, RF achieved relatively good overall performance; after historical environmental information was added, Deep Forest performed better under E2, E3, and E4. This result indicates that, under the current dataset and spatially grouped validation framework, different models have different adaptability to multi-temporal environmental features.

This result also suggests that model performance depends not only on the algorithm itself but also on the temporal scale of the input features. For relatively simple instantaneous feature sets such as E1, RF was already able to use the main environmental variable information effectively. When 7-day or 30-day rolling statistics were added, the temporal associations and potential redundancy among input variables increased, and the model required stronger multilevel feature-integration ability. The cascade forest structure of Deep Forest can gradually combine original variables and outputs from previous layers and may be more suitable for representing complex relationships among multi-variable and multi-temporal environmental information. He et al. [38] also noted that the multilayer structure of Deep Forest can reflect changes in feature contributions across different layers.

Previous fishery-prediction studies have shown that machine-learning models can be used for fish habitat or fishing-ground distribution prediction and can be combined with SHAP to identify key predictive variables and their contributions to model output. Zhang et al. [39] used machine-learning models and SHAP to analyze the contributions of environmental variables in albacore fishing-ground prediction. Shi et al. [40] used three-dimensional marine environmental variables and interpretable machine learning to predict yellowfin tuna fishing grounds. These studies indicate that environmental variable type and ecological background can affect model interpretation. Together with the results of this study, this suggests that the adaptability between model structure and input-feature temporal scale may also influence prediction performance.

### 4.3. Limitations and Future Perspectives

Although this study constructed 7-day and 30-day features to represent short-term environmental fluctuations and long-term background effects, lag analysis showed that some variables, such as DO and CV, reached correlation peaks within 12–22 days. This suggests that an intermediate response scale may exist between the short- and long-term windows. Because this study used discrete temporal windows for feature construction, the potential role of this intermediate scale may not have been fully captured. Future studies could introduce finer temporal windows, such as 14-day or 15-day rolling windows, to further evaluate environmental responses along a continuous temporal dimension.

Regarding the classification of high-catch and low-catch samples, this study used the median daily catch as the threshold, which helped construct a relatively balanced binary classification task. However, this threshold only represents a relatively high-catch state within the current dataset and was not systematically compared with other quantile thresholds or year-specific thresholds; therefore, threshold-sensitivity analysis is still needed in future studies. Meanwhile, commercial catch may be affected by changes in fishing effort. Changes in vessel numbers and fishing intensity among years may increase the probability of high-catch records, but they do not necessarily indicate a synchronous increase in resource abundance. Therefore, the results of this study should be interpreted as predictions of catch-based high-catch fishing grounds rather than direct estimates of absolute resource abundance. Future studies could further incorporate standardized CPUE, fishing days, sets, vessel numbers, vessel type, and gear differences to reduce the bias caused by changes in fishing effort.

The environmental variables used in this study mainly represent sea-surface and upper-ocean conditions, and three-dimensional environmental features such as vertical temperature, vertical DO, thermocline structure, and vertical water-mass variation were not fully incorporated. For pelagic migratory species such as Chub Mackerel, vertical environmental structure may affect habitat depth, feeding activity, and spatial distribution. In addition, spatial aggregation of fishery records and environmental data helps unify data scales. Future studies could combine higher-resolution three-dimensional environmental data, ocean-dynamic information, and more detailed fishing-position records to more comprehensively characterize fishing-ground environmental conditions.

Different models also performed differently under different temporal-scale feature conditions. Therefore, model selection should consider not only general algorithm performance but also the temporal attributes of environmental features, the ecological association characteristics of the target species, and the specific prediction task. Future studies could conduct ablation experiments for predictive interpretation and compare model stability under continuous temporal features, vertical environmental variables, and external validation datasets, thereby improving model applicability and generalization.

### 4.4. Practical Implications

The results of this study may provide a reference for Chub Mackerel fishing-ground prediction and fishery management. Short-term environmental changes may have value for high-catch fishing-ground prediction. In particular, 7-day rolling environmental features help reflect recent marine environmental states and can theoretically be updated daily using remote-sensing and reanalysis data. Therefore, in practical applications, 7-day rolling statistics of variables such as SST, SSS, Chl-a, DO, and MLD could be used as dynamic inputs in near-real-time fishing-ground prediction systems to help generate preliminary estimates of short-term high-catch probability or potentially suitable fishing areas. At the same time, 30-day rolling variables can provide monthly-scale environmental background information. When combined with 7-day rolling variables, they can help the model simultaneously use recent environmental changes and longer-term environmental conditions. The results of this study show that multi-temporal-scale features had relatively good numerical performance in high-catch sample identification, indicating that the fusion of environmental information at different temporal scales can provide a more complete environmental representation for fishing-ground prediction. In terms of practical application, this study may provide auxiliary reference for fishers in selecting operation areas and planning fishing trips, helping reduce ineffective searching and unnecessary fuel consumption. It can also be used to track the spatiotemporal variation in high-catch fishing grounds and provide information support for fishery forecasting, dynamic management of fishing activities, and resource monitoring. It should be emphasized that this study predicts the high-catch state defined based on fishing records, rather than resource abundance or population biomass. Therefore, model outputs should be interpreted together with fishing effort, management systems, stock-assessment results, and field experience, so as to avoid directly equating prediction results with changes in resource abundance.

## 5. Conclusions

This study used fishery data for Chub Mackerel in the Northwest Pacific from 2014 to 2022 to evaluate the effects of environmental features at different temporal scales on high-catch fishing-ground prediction for Chub Mackerel (defined based on catch), and SHAP was used to analyze the contributions of environmental variables to model prediction. The results showed that historical environmental information can supplement instantaneous environmental variables to some extent and help improve the model prediction ability for high-catch fishing grounds; therefore, the hypothesis proposed in this study was generally supported. The main conclusions are as follows:

(1) Environmental features at different temporal scales affected the prediction of high-catch fishing grounds for Chub Mackerel. Short-term environmental features helped improve the overall discrimination ability of the model, whereas multi-scale feature fusion further enhanced high-catch sample identification.
(2) Lag analysis and SHAP results indicated that model prediction did not rely on a single variable, but jointly used multi-temporal environmental information, including Chl-a, SST, SSS, DO, MLD, and their rolling statistics. Short-term variables mainly reflected recent environmental-change information, long-term variables provided background conditions, and seasonal signals also made certain contributions.
(3) In practical applications, operational fishing-ground prediction systems are recommended to incorporate 7-day rolling environmental features to improve short-term fishing-ground discrimination. When the objective focuses more on identifying high-catch areas, 7-day and 30-day environmental features can be further integrated. Future studies could combine fishing-effort information, standardized CPUE, and three-dimensional environmental variables to improve model robustness and application value.

## Figures and Tables

**Table 1:** Composition and ecological meaning of different temporal-scale feature sets.

Feature Set	Temporal Scale	Feature Composition
E1	Instantaneous scale	Base environmental variables + seasonal cyclic encoding (month_sin, month_cos)
E3	Short-term scale	E1 + 7-day rolling means
E4	Long-term scale	E1 + 30-day rolling means
E2	Multi-temporal scale fusion	E1 + 7-day + 30-day rolling means

**Table 2:** Representative hyperparameter combinations with the highest inner-validation AUC for each model.

Model	Feature Set	n_Candidates	Best Inner AUC	Optimal Hyperparameters
Deep Forest	E2	10	0.8215	max_depth = 10
				max_features = sqrt
				max_layers = 4
				min_samples_leaf = 5
				n_estimators = 2
				n_trees = 100
RF	E2	10	0.8166	max_depth = unlimited
				max_features = sqrt
				min_samples_leaf = 3
				n_estimators = 100
XGB	E2	10	0.8097	colsample_bytree = 0.8
				learning_rate = 0.1
				max_depth = 5
				n_estimators = 200
				subsample = 0.8
LGBM	E3	10	0.8077	bagging_fraction = 0.8
				feature_fraction = 0.8
				learning_rate = 0.1
				n_estimators = 200
				num_leaves = 31
CAT	E2	10	0.8301	depth = 6
				iterations = 200
				l2_leaf_reg = 3
				learning_rate = 0.03

**Table 3:** Prediction performance of each model under different temporal-scale feature sets.

Model	E_Set	AUC	F1	Precision	Recall	PR-AUC
DF	E1	0.7041	0.6393	0.6424	0.6537	0.6673
DF	E2	0.8199	0.7513	0.7675	0.7729	0.7988
DF	E3	0.8265	0.6870	0.6919	0.7190	0.7844
DF	E4	0.8185	0.6645	0.7049	0.6551	0.7394
CAT	E1	0.7235	0.6230	0.6318	0.6422	0.6927
CAT	E2	0.8192	0.7370	0.7489	0.7515	0.8066
CAT	E3	0.8264	0.6899	0.7034	0.6946	0.7719
CAT	E4	0.8091	0.6528	0.6867	0.6429	0.7425
LGBM	E1	0.6960	0.6297	0.6066	0.6649	0.6531
LGBM	E2	0.8062	0.6884	0.7036	0.7067	0.7496
LGBM	E3	0.8016	0.6741	0.6757	0.6964	0.7456
LGBM	E4	0.7650	0.5907	0.5985	0.6096	0.6969
RF	E1	0.7082	0.6499	0.6432	0.6824	0.6731
RF	E2	0.8373	0.6923	0.7424	0.6966	0.7738
RF	E3	0.8219	0.6756	0.6996	0.7017	0.7666
RF	E4	0.7953	0.6164	0.6820	0.6002	0.7103
XGB	E1	0.7172	0.6260	0.6127	0.6612	0.6911
XGB	E2	0.8021	0.7056	0.7143	0.7256	0.7885
XGB	E3	0.8210	0.6979	0.7095	0.7095	0.7673
XGB	E4	0.7900	0.6038	0.6523	0.5925	0.7114

**Table 4:** Models with relatively better overall performance and their prediction performance under each temporal-scale feature set.

Feature Set	Best Model	AUC	F1-Score	Precision	Recall	PR-AUC
E1	RF	0.7082	0.6499	0.6432	0.6824	0.6731
E2	DF	0.8199	0.7513	0.7675	0.7729	0.7988
E3	DF	0.8265	0.6870	0.6919	0.7190	0.7844
E4	DF	0.8185	0.6645	0.7049	0.6551	0.7394

**Table 5:** Time-lag correlation results between environmental variables and high-catch fishing grounds for Chub Mackerel.

Variable	Best Lag	Spearman’s ρ	*p*-Value	FDR *p*-Value	95% CI
CV	20 days	−0.3704	0.00330	0.02243	[−0.5692, −0.1308]
DO	14 days	0.4047	0.000683	0.01002	[0.1822, 0.5877]
MLD	2 days	−0.5561	1.04 × 10^−7^	2.89 × 10^−5^	[−0.6921, −0.3819]
Chl-a	1 day	−0.4695	1.45 × 10^−5^	0.000705	[−0.6266, −0.2758]
SLA	24 days	−0.3458	0.00842	0.03982	[−0.5563, −0.0937]
SSS	1 day	−0.5154	1.00 × 10^−6^	0.000104	[−0.6603, −0.3334]
SST	30 days	0.4198	0.00216	0.01753	[0.1631, 0.6233]
UGOS	25 days	0.2649	0.04852	0.13538	[0.0021, 0.4934]
VGOS	25 days	0.3502	0.00814	0.03934	[0.0962, 0.5614]

## Data Availability

The fisheries logbook data used in this study are not publicly available due to copyright and commercial confidentiality restrictions. Access to the data may be granted by the corresponding author upon reasonable request.
